# A multiobjective migration algorithm as a resource consolidation strategy in cloud computing

**DOI:** 10.1371/journal.pone.0211729

**Published:** 2019-02-06

**Authors:** Danqing Feng, Zhibo Wu, DeCheng Zuo, Zhan Zhang

**Affiliations:** 1 Computer Science and Technology, Harbin Institute of Technology, Harbin, China; 2 Computer Science and Technology, Air Force Communication NCO Academy, DaLian, China; Chongqing Jiaotong University, CHINA

## Abstract

To flexibly meet users’ demands in cloud computing, it is essential for providers to establish the efficient virtual mapping in datacenters. Accordingly, virtualization has become a key aspect of cloud computing. It is possible to consolidate resources based on the single objective of reducing energy consumption. However, it is challenging for the provider to consolidate resources efficiently based on a multiobjective optimization strategy. In this paper, we present a novel migration algorithm to consolidate resources adaptively using a two-level scheduling algorithm. First, we propose the grey relational analysis (GRA) and technique for order preference by similarity to the ideal solution (TOPSIS) policy to simultaneously determine the hotspots by the main selected factors, including the CPU and the memory. Second, a two-level hybrid heuristic algorithm is designed to consolidate resources in order to reduce costs and energy consumption, mainly depending on the PSO and ACO algorithms. The improved PSO can determine the migrating VMs quickly, and the proposed ACO can locate the positions. Extensive experiments demonstrate that the two-level scheduling algorithm performs the consolidation strategy efficiently during the dynamic allocation process.

## Introduction

Cloud computing is considered one of the most promising technologies to meet customer demand flexibly. Usually, it includes SaaS, PaaS, and IaaS. Software as a service (SaaS) provides access to complete applications as a service [1], and platform as a service (PaaS) provides a platform to develop other applications, such as the Google App Engine (GAE) [2]. Infrastructure as a Service (IaaS) [3–4] provides an environment to deploy the managed virtual machines. A reasonable resource allocation strategy can help to consolidate resources and reduce energy consumption. From the perspective of the providers, the key issue to be solved is to maximize the utilization by reducing the fundamental costs. As a core technique, virtualization [5–7] provides an effective way to pack the application requests into the VMs. The virtualization technique can make full use of the utilization by decreasing the power consumption. Virtual mapping [8] has become one of the core techniques in datacenters, which provides a solution to the resource allocation. Generally, the problems to be solved are divided into two subproblems: when to migrate and where to locate.

Traditionally, researchers have focused more on energy consumption with the single objective of CPU utilization. The VM placement problem is usually solved by the bin-packing algorithm, which is an NP-hard problem [9–10]. For example, the pMapper system [11] proposed to determine the power-cost trade-offs by minimizing the costs with the minimum number of machines using the improved FFD algorithm. Another approach is best fit decreasing (BFD). Researchers proposed modified best fit decreasing (MBFD), which is an extension of the BFD method to improve energy efficiency under dynamic workloads [12]. However, these bin-packing algorithms focus on improving the energy efficiency but ignore other elements, such as service level agreement (SLA) violation and resource wastage, which have impacts on the dynamic scheduling process. However, additional elements would make the bin-packing algorithm more complex. Most researchers focus on heuristic algorithms to solve dynamic scheduling problems. For example, the simulated annealing virtual machine placement (SAVMP) approach further improves of first fit (FF) algorithm, which minimizes the total power consumption in the datacenter [13]. It reduces the energy consumption based on CPU utilization; however, other factors should also be focused, such as the CPU, the memory. In [14], the genetic algorithm is used to reconfigure resources to minimize the migration cost, which has the advantage of proposing a weight function that includes the CPU and the memory. However, the migration cost function always takes the memory as the only optimization objective. The modified PSO (MPSO) algorithm [15] was introduced to improve the energy efficiency of the CPU and the disk. It makes use of the utilization and reduces the number of VM migrations. To avoid falling into a local search, several authors have proposed two-phase mechanisms to solve the optimization problem. In [16], the GA-ACO algorithm was proposed to improve the performance. The GA algorithm is used for the local search, and the ACO algorithm is used to escape the local search to improve the search performance. The proposed GA-ACO algorithm improved the performance effectively, but it considered fewer factors, such as the performance. In [17], the optimization model was used to minimize the total cost. The proposed ACO and GA algorithms are used in the global and local searches. However, from the perspective of the providers, more elements should be considered during the scheduling process, such as maximizing CPU utilization and memory utilization.

**Fig 1:**
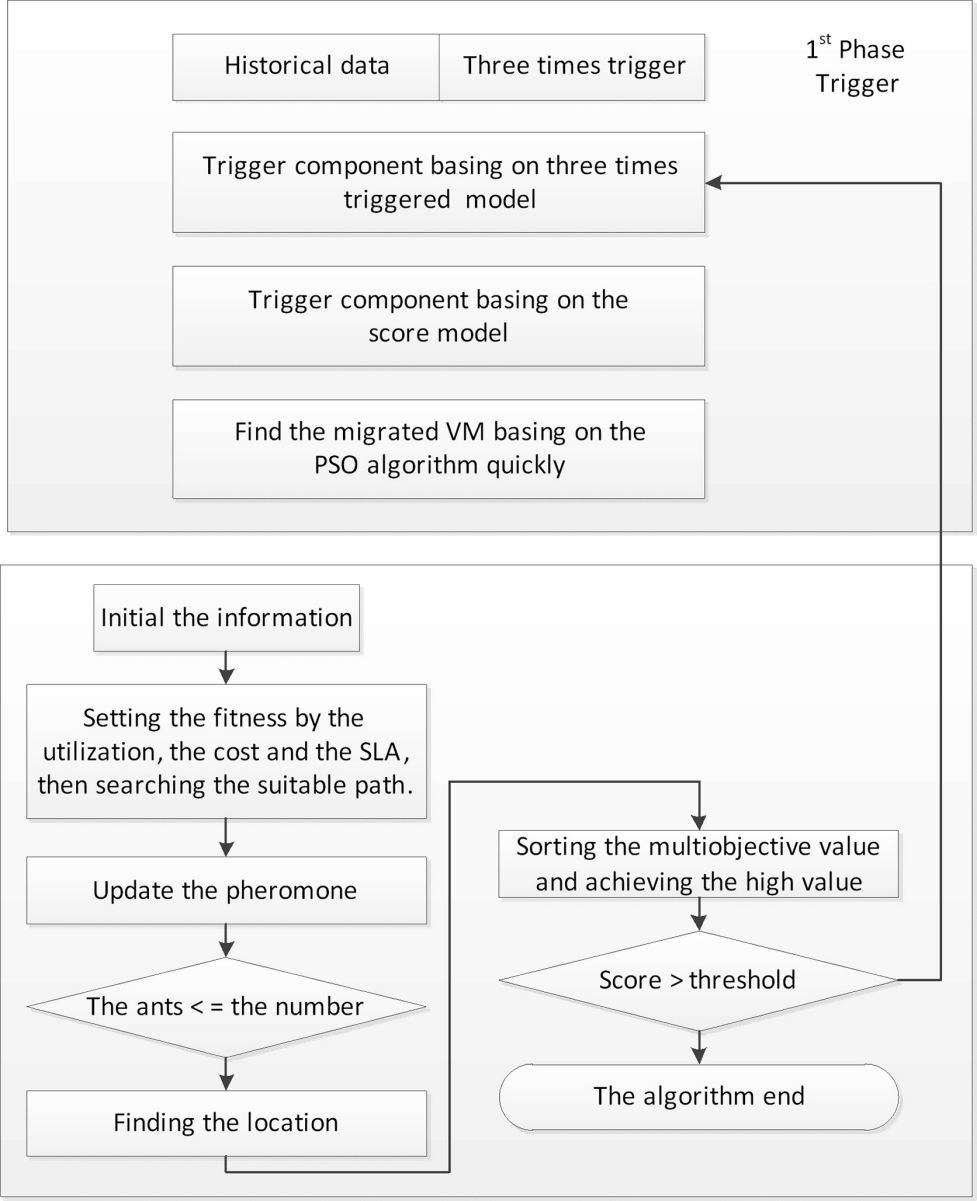
Two-level hybrid heuristic algorithm.

In summary, many researchers have focused on improving energy efficiency [18–20]. However, few studies have investigated solving the multiobjective optimization problem. Providers should consider additional factors, such as reducing the power consumption, maximizing the utilization, and avoiding SLA violations. Hence, we present a two-level algorithm to achieve lower costs and power consumption, which is shown in Fig 1. The first phase determines the hotspots by using a proposed score model and then migrates the VMs by using the PSO algorithm. The second phase finds the locations by using the improved ACO algorithm. Generally, the proposed algorithm aims to maximize the utilization and minimize the energy consumption. The contributions in this paper are as follows.

- First, the proposed method solves the issue of when to migrate. We propose a score model to determine the hotspots by using the GRA and TOPSIS methods, which simultaneously considers multiple objectives, such as CPU utilization and memory utilization.
- Second, this method migrates the VMs quickly. In this phase, we use the improved PSO algorithm to find the VMs to migrate by considering both CPU utilization and memory utilization. The PSO algorithm obtains the results quickly [21].
- Thirdly, this method solves the issue of where to migrate. It locates the suitable positions to reduce the rental costs, SLA violations and power consumption by using the consolidation strategy, which is implemented by the proposed Ant Colony Optimization (SACO) algorithm.

The proposed approach improves the performance by simultaneously minimizing the rental cost and reducing the power consumption. The remainder of this paper is organized as follows. Section 2 presents the research motivation and the current related studies of solving the dynamic scheduling problem. Section 3 provides the architecture of the resource optimization and analyzes the scheduling phases in cloud computing, and Section 4 presents the two-level hybrid scheduling algorithm in detail. Section 5 presents the results of the extensive experiments and makes a compares them with the approaches described above. Finally, Section 6 provides the conclusions and describes future work.

## Related work

### Dynamic scheduling strategy

Previous studies on resource consolidation strategies are divided into three main categories: static strategies [22–23], dynamic scheduling [24–26] and decision-making on the prediction [27–29]. The traditional static approach can be implemented to meet a varying demand, but it generates more overheads Halder et al. [30] presented a static consolidating algorithm that considered CPU utilization and SLA violations. Tiago et al. [31] proposed an LP and heuristics method to complete the mapping from the VMs to the host, which would reduce the number of migrations with the minimum penalty. The disadvantage of the static consolidation strategies is that they cause resource wastage to meet sudden load demands. An example of a dynamic scheduling strategy is the depending on the prediction technique. Cloudscale [32] achieved adaptive resource allocation with lower resource and energy costs by integrating VM resources with dynamic voltage and frequency scaling (DVFS) to save energy. Press [33] proposed the fine-grained mechanism, which reduces the resource wastage and SLO violations. However, the predictive technique is complex, and it is difficult to obtain accurate results with this technique. In dynamic scheduling strategies, the issue to be solved is when and where to migrate. Lovász et al. [34] presented a dynamic strategy that uses the greedy and modified first-fit algorithm and considers the power and response time as the performance metrics. Seyed et al. [35] proposed an adaptive threshold-based algorithm to detect overloaded hosts, which considered the optimization based on the energy performance trade-off. The best method is to determine the adaptive threshold by learning, but the disadvantage is that it focuses on the energy consumption; additional elements have effects during the dynamic scheduling process, such as the SLA and migration cost.

Most studies have focused more on the single objective of minimizing energy consumption. However, more factors should be considered during the scheduling process. For example, providers also emphasize maximizing the utilization, including the CPU and the memory. In addition, to reduce SLA violations, the proposed two-level method reduces energy and resource wastage.

### Multiobjective optimization

One of the most important factors in server consolidation algorithms is the energy consumption. However, additional factors (e.g., the cost overhead, memory utilization, and SLA violations) should also be considered in the optimization algorithm. For example, Leili et al. [12] proposed an adaptive fuzzy threshold to detect overloaded or underloaded thresholds. The advantage of the method is that it proposes a double adaptive threshold to determine when the migration starts or where the VMs migrate. However, the proposed approach uses the energy and performance as the evaluation metrics. The MISTRAL [36] architecture proposed a strategy to reduce the power consumption and adaptation cost, which implemented the cost decision-making based on the response time. However, it developed a control architecture to solve the power trade-offs rather than several objectives. In [37], a multiple objective ant colony system algorithm that focuses on two objectives, including the makespan and the user’s budget, was presented. This strategy had the advantage of minimizing the rental costs more efficiently; however, it focused more on maximizing the resource utilization. Additionally, it ignored other elements, such as the SLA and resource wastage. In [38], the genetic algorithm was proposed to enhance the system provisioning, system performances, system failure and network overheads. The method considered more factors than the previous methods, but it ignored the cost overheads. In [39], the CMCVRP optimization model was presented to reduce costs and energy consumption. The method used cost reduction model to achieve the cost reduction percentages. In [40], the proposed method allocated the resources to minimize the total amount of resources while meeting the end-to-end performance requirements for the application. The method described in [41] provided a theoretical control solution to the dynamic capacity provisioning problem that minimizes the total energy cost while meeting the performance objective of task scheduling delays. The response time and the temperature also play important roles in data centers, which can greatly affect the service quality.

Based on the studies presented above, Table 1 provides a list of several consolidation algorithms that are used to solve optimization problems. The main optimization parameters are the minimization of the energy overheads and the cost overhead and the maximization of the application performance. We propose a two-level hybrid algorithm. First, the algorithm determines when to migrate. The proposed score model based on the GRA and TOPSIS methods achieves the adaptive threshold depending on the CPU utilization and memory utilization. Second, the hybrid heuristic algorithm includes the PSO and ACO algorithms, which emphasizes reducing SLA violations and the energy consumption. In addition, the PSO algorithm determines the hotspot VMs quickly, and the proposed ACO algorithm focuses on solving the VM placement problem to reduce SLA violations and the energy consumption.

**Table 1:** Comparison of the consolidation algorithms.

Paper	Decision	Performance	SLA	Energy	Overheads	Utilization
[30]	Static	No	Yes	No	No	Yes
[31]	Static	No	Yes	No	No	No
[32]	Prediction	No	No	Yes	Yes	No
[33]	Prediction	No	Yes	No	Yes	No
[35]	Dynamic	Yes	No	Yes	No	No
[12]	Multiobjective	Yes	No	Yes	No	No
[36]	Multiobjective	No	No	Yes	Yes	No
[37]	Multiobjective	No	No	No	Yes	Yes
[38]	Multiobjective	Yes	No	No	Yes	No
[39]	Multiobjective	No	No	Yes	Yes	No
[40]	Multiobjective	Yes	No	No	Yes	No
[41]	Multiobjective	Yes	No	Yes	Yes	No

## Background

### Description of the main components

**Fig 2:**
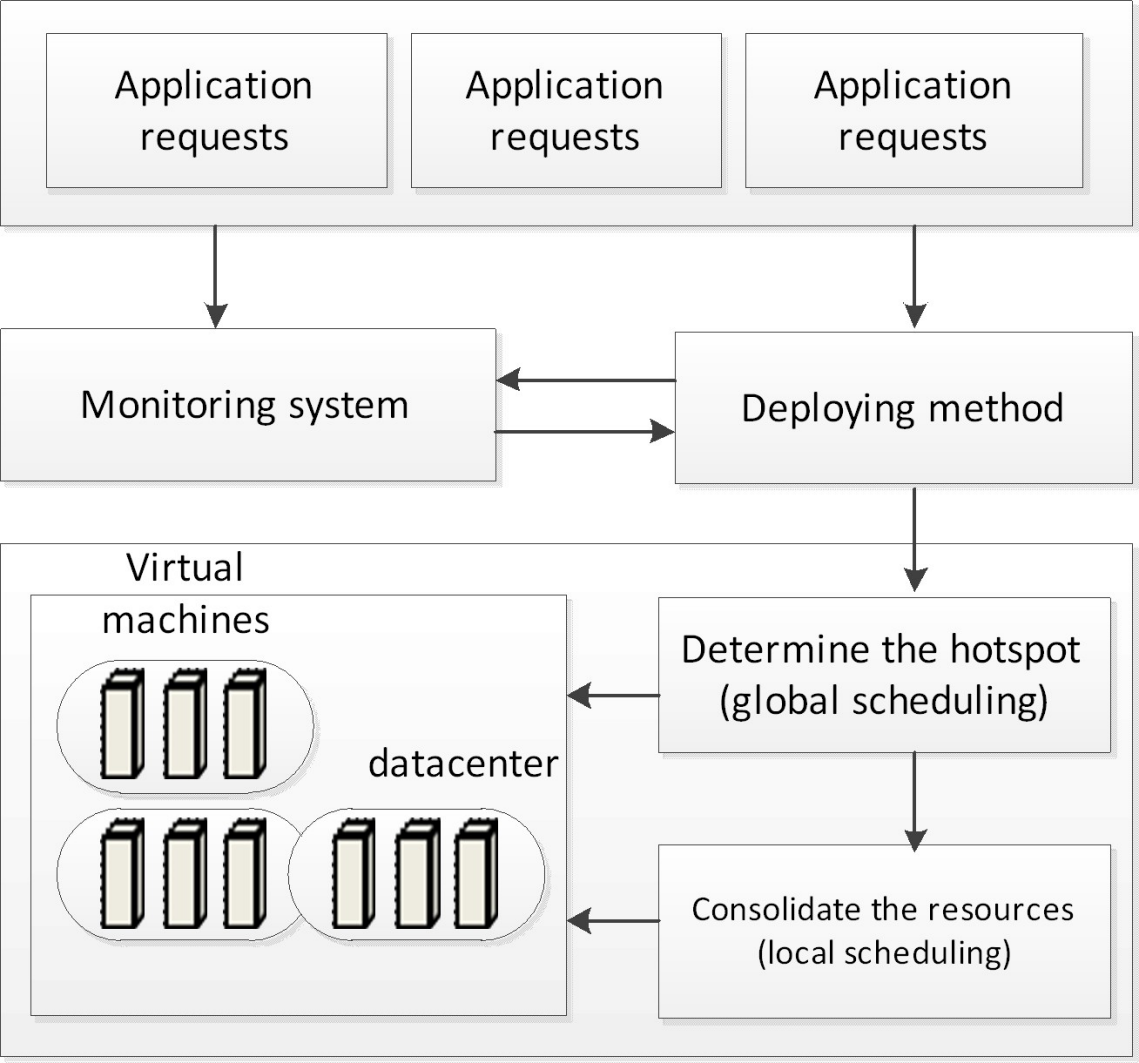
The main components of the architecture.

We propose a two-phase algorithm to conduct resource allocation in datacenters for cloud computing. The designed architecture is shown in Fig 2. When the application requests arrive, the monitoring system collects the data to calculate the threshold. We then take the threshold as the baseline to efficiently manage and consolidate the resources. The architecture includes a local scheduling mechanism and a global scheduling mechanism. The global scheduling mechanism determines the hotspots by using the score model based on the GRA and TOPSIS methods, and the local scheduling mechanism efficiently solves the VM placement problem. The main components are described in detail as follows.

- Application request

In cloud computing, it is essential to meet the users’ demands at any time. Before the requests are executed, the providers provision the resources rapidly to meet the users’ demands. The goal of the providers is to reduce the rental cost by maximizing the utilization and minimizing the energy consumption during the scheduling process.

- Monitoring system

The monitoring system collects the data, including the CPU utilization and the memory utilization. The collected information is used to calculate the score threshold by using the proposed model, which is described in detail in the next section. When the workload reaches the specified value, the proposed algorithm implements the efficient resource allocation method during the dynamic scheduling process.

- Global scheduling

The global scheduling mechanism includes three parts: the triggering part, selection part and location part. First, the triggering part determines the overloaded hosts and solves the issue of when to migrate. The selection part then determines which virtual machines are to be migrated. Finally, the location part solves the issue of where to migrate.

- Local scheduling

The local scheduling mechanism solves the VM placement problem by using the improved ACO algorithm. In addition, the proposed ACO algorithm emphasizes maximizing the utilization, avoiding SLA violations and reducing the power consumption.

### The score threshold for hotspot detection

In this section, we present the score model based on the GRA and TOPSIS methods to determine the hotspots. The upper score threshold is triggered when the hotspot hosts have been located. The score threshold rule takes the GRA and TOPSIS methods as the multiobjective policy considering four metrics, as shown in Table 2. The policy scores every PM, including the CPU utilization and the memory utilization. It also considers two criteria, cost criteria and benefit criteria. For the cost criteria, we obtain a positive solution when the value is smaller. This is opposite to the benefit criteria. Furthermore, it is necessary to select the PM with more VMs considering cost and benefit, which likely reduces the rental cost. Based on the proposed policy, the selected hotspots are those that exceed the upper score threshold. The policy is described in detail below.

**Table 2:** The main parameters of the scheduling process.

Parameter	Description	Type
CPU Cycle	CPU Clock Speed	Cost
Spare Memory	The rest memory in the PM	Cost
CPU%	The utilization of CPU in the PM	Benefit
MEM%	The utilization of memory in the PM	Benefit

Normalization of the decision matrix: By using the Delphi method [42], it is easier to determine the key factors, such as the CPU cycle, spare memory, CPU utilization and memory utilization, which are shown in Table 2. Because the data are different in the experiments, the normalization method uses the average in each column to normalize the decision matrix R, which is listed in Eq 1.

$$R=\left[\begin{array}{ccc} r_{11} & \cdots & r_{1n}\\ \vdots & \cdots & \vdots \\ r_{m1} & \cdots & r_{mn} \end{array}\right]$$ (1)

Improved TOPSIS: TOPSIS is the abbreviation for the technique for order preference by similarity to ideal solution [43]. The improved ideal solutions determine the positive benefit or cost criteria as listed in Eq 2. The negative value is determined using Eq 3.

$$P_{j}^{+}=\{(\max({\tilde{r}}_{ij})|i\in I),\left(\min\left({\tilde{r}}_{ij}\right)|i\in J\right)\}$$ (2)

$$P_{j}^{-}=\left\{\left(\min\left({\tilde{r}}_{ij}\right)|i\in I\right),\left(\max\left({\tilde{r}}_{ij}\right)|i\in J\right)\right\}$$ (3)

Grey Relational Analysis: Grey theory [44] is an effective way to explore system behavior using limited information. We describe the GRA method in detail below. First, we determine the difference between the comparative series *r*_*jk*_ and the standard series $P_{k}^{+}$ or $P_{k}^{-}$. The distinguished coefficient *ρ* usually has a value of 0.5 and is generally between [0, 1]. The Grey relational coefficients *ς*^+^and *ς*^−^ are determined using Eq 4 and Eq 5, respectively.

$$\varsigma^{+}(k)=\frac{\underset{j}{\min}\;\underset{k}{\min}|r_{jk}-P_{k}^{+}|+\rho \underset{j}{\max}\;\underset{k}{\max}|r_{jk}-P_{k}^{+}|}{|r_{jk}-P_{k}^{+}|+\rho \underset{j}{\max}\;\underset{k}{\max}|r_{jk}-P_{k}^{+}|}$$ (4)

$$\varsigma^{-}(k)=\frac{\underset{j}{\min}\;\underset{k}{\min}|r_{jk}-P_{k}^{-}|+\rho \underset{j}{\max}\;\underset{k}{\max}|r_{jk}-P_{k}^{-}|}{|r_{jk}-P_{k}^{-}|+\rho \underset{j}{\max}\;\underset{k}{\max}|r_{jk}-P_{k}^{-}|}$$ (5)

We then determine the degree of relation *r* on the weight coefficients *ω* multiplied by the Grey relational coefficient *ς*(*k*). The weight coefficients are determined by the analytic hierarchy process (AHP) method [45–46]; they are respectively 0.19, 0.28, 0.29 and 0.24. The degrees of relation *r*^+^ and *r*^−^ are calculated using Eqs 6 and 7, respectively.

$$r^{+}=\sum_{k=1}^{m}\omega_{k}\varsigma_{}^{+}(k)$$ (6)

$$r^{-}=\sum_{k=1}^{m}\omega_{k}\varsigma_{}^{-}(k)$$ (7)

Relative Closeness: Based on the analysis presented above, the ideal solution determines the related closeness *d*^+^ using Eq 8. A higher value indicates that it is closer to the positive solution, and a lower value is closer to the negative solution. The negative related closeness *d*^−^ is calculated using Eq 9.

$$d^{+}=\frac{r^{-}}{r^{+}+r^{-}}$$ (8)

$$d^{-}=\frac{r^{-}}{r^{+}+r^{-}}$$ (9)

Score Model: A server might be overloaded. Multiple objectives should be involved in the scheduling process; the objectives are shown in Table 2. In this paper, we take the GRA and TOPSIS methods as the decision strategy to determine the hotspots. The overloaded score threshold is described by Eq 10, and it represents the point at which when to migrate. The parameter *U*_*t*_ represents the CPU utilization of the host, and *d*^+^ and *d*^−^ are the positive related closeness and the negative related closeness, respectively. Similarly, when the score is higher, the PM can easily show the hotspots.

$$Score^{+}=\frac{d^{+}\times U_{t}}{d^{+}\times U_{t}+d^{-}\times (1-U_{t})}$$ (10)

## Proposed structure

In this section, we propose a two-level scheduling algorithm aimed at maximizing the utilization, avoiding SLA violations and reducing the energy consumption during the scheduling process. We divide the consolidating algorithm into three parts: the triggering part, selection part and location part. These parts are described in detail as follows. In the triggering part, we determine the hotspots by the score model. It describes the time at which the overloaded PMs migrate. In the selection part, we select the VMs from the overloaded and under-loaded PMs. Besides, we also quickly select the VMs from the hotspots by the PSO. In the location part, we place the migrated VMs into the selected positions by the improved Ant Colony Optimization (ACO) algorithm. The hybrid algorithm is shown as Algorithm 1. We provide a detailed description below.

**Algorithm 1: The hybrid two-level heuristic algorithm**

**Input**: VM_list and PM_list

**Output**: Migration_list

1. Monitor the PMs and score every PM

2. Score the PM_list by using the proposed score model, and divide the PMs into overloaded PMs or under-loaded PMs

3. Choose the highest VM according to the highest distance formula (15) in the overloaded PMs by using the PSO algorithm

4. Choose all of the VMs in the underloaded PMs

5. Obtain the Migration_list of the VMs by using Step 3 and 4

6. Place the selected VMs to determine the reasonable locations by using the improved ACO algorithm based on multiobjective optimized model

7. Return the score model

### Hybrid algorithm

The hybrid algorithm is a two-level hybrid algorithm. *The first level* includes a triggering part and a selection part, and it aims at determining the threshold and the migrated VMs. In addition, the selected VMs are quickly identified by the PSO algorithm. *The second level* includes the location part to solve the VM placement problem, which is implemented by the ACO algorithm.

In the first level, during the scheduling process, we obtain the score threshold as the triggering threshold by using the monitoring mechanism. In this phase, we consider both the CPU and the memory as the key factors to determine the source and target host machines under the fluctuating workload. If the demands exceed the triggering threshold, more VMs are provided to supply the demand at any time.

Triggered threshold: The triggering threshold is determined by the score model listed in Eq 10. In general, this host would be considered to be overloaded when the current utilization exceeds the upper threshold. In the experiments, the upper utilization threshold is usually set to 0.8. When the value exceeds the upper threshold, the servers are scaled up to meet the demand. The lower utilization threshold is usually set to 0.2, which is determined by experiments. We then define the score threshold by Theorem 1 and Lemma 1, which are shown as below. Hence, we determine that the triggered score threshold is greater than 80 percent, which mostly depends on the CPU utilization *U*_*t*_ and the related closeness *d*.

**Theorem 1**. The upper CPU threshold is 0.8 in most experiments. Therefore, during the dynamic scheduling process, the upper score threshold is higher than 80 percent.

Proof.

First, we take the inverse of Eq 10 on both sides at the same time and obtain Eq 11.

$$\frac{1}{Score^{+}}=\frac{d^{+}\times U_{t}+d^{-}\times (1-U_{t})}{d^{+}\times U_{t}}$$ (11)

A utilization greater than 0.8 indicates an overloaded state. During the experiments, we find that *d*^+^ and *d*^−^ are 0.49 and 0.51, respectively. They are approximately 0.5 in the dynamic process. We start the calculation with Eq 12. By taking the inverse again, we determine that the hotspot score threshold is 80 percent, as shown in Eq 13.

$$\frac{1}{Score^{+}}\leq \frac{0.5\times 0.8+0.5\times 0.2}{0.5\times 0.8}=\frac{4+1}{4}$$ (12)

$$\Rightarrow Score^{+}\geq \frac{4}{5}$$ (13)

**Lemma 1**. In the overloaded state, the upper score threshold is greater than 80 percent based on Theorem 1. Formally, the lower score threshold is (1−*Score*^+^), which is less than 20 percent.

Proof.

The lower score model is listed as Eq 14.

$$Score^{-}=(1-Score^{+})$$ (14)

Generally, the uppper threshold arg min(*Score*^+^) is achieved, and it is approximately 80 percent in the over provisioning in Theorem 1. We then determine that the lower score arg max(*Score*^−^) is nearly 20 percent in the under provisioning.

Selection part: By determining the triggering threshold, we can select several VMs to migrate. In the experiments, we find that the CPU more easier reaches a specific value than the memory. Hence, we use the Euclidean distance method [47] to construct a distance model that includes both CPU utilization and memory utilization, as described in Eq 15. The selection policy selects the VM with the greatest distance to migrate by the PSO algorithm to avoid the overloaded state. The PSO algorithm is described in detail below. In addition, to reduce the energy consumption, we select all of the VMs hosted in the underloaded PMs to migrate.

- Step 1: Initialize all of the particles. The selection starts in the particles randomly.
- Step 2: For each particle, the fitness value is calculated using Eq 15, which includes the CPU utilization and memory utilization. If the fitness value is greater than the set value, we set the current value as *pBest* in the local search.

$$dis\tan ce=\sqrt{{\left(CPU\%\right)}^{2}+{\left(MEM\%\right)}^{2}}$$ (15)

- Step 3: Choose the particle with the greatest fitness value of the particles as *gBest*. We then finish the global search.
- Step 4: Update the velocity and the position using Eqs 16 and 17, respectively. In Eqs 16 and 17, *v* is the velocity, *present* is the position, and. *c*_1_ and *c*_2_ are learning factor, which are equal to 2 in the traditional PSO algorithm [48].

$$v=w\times v+c_{1}\times rand\times (pBest-present)+c_{2}\times rand\times (gBest-present)$$ (16)

present=present+v (17)

- Step 5: Verify the number of iterations. If the number of iterations exceeds the maximum, the cycle is terminated.

In the second phase, the emphasis is on solving the VM placement problem. Previous researchers have focused more on the energy consumption. We propose a solution to obtain the reasonable positions depending on multiple objectives, including the SLA violations, resource wastage and energy consumption. These objectives are described in detail below.

SLA: The SLA violations are calculated as the difference between the actual requests and the allocated requests (AR) divided by the total requests (TR), as shown in Eq 18. That is, the SLA is defined as the ratio of difference requests to total requests. Eq 18 is described as follows.

$$SLA=\frac{TR-AR}{TR}$$ (18)

Resource Wastage: To make full use of the resources, we consider the CPU and the memory to define the resource wastage as in Eq 19.When the resource wastage *W* is large, more resources are wasted. In Eq 19, *U*^*P*^ is the CPU utilization, and *U*^*m*^ is the memory utilization. Eq 19 aims to make full of the resources depending on the CPU and the memory.

$$W=\frac{|U^{p}-U^{m}|}{\sqrt{{\left(U^{p}\right)}^{2}+{\left(U^{m}\right)}^{2}}}$$ (19)

Energy consumption: Previous studies have shown that idle servers consume approximately 70 percent of their peak power [49]. The energy consumption is given by Eq 20. We perform the experiments using Inspur Servers, which consume 700 W at full utilization. The idle power consumption coefficient *k* is equal to 0.7, and the current host power consumption ranges from the idle state (490 W) to full utilization (700 W). *P*_max_ represents the peak power, which is equal to 700 W in the experiments, and *u* is the CPU utilization.

$$P=k\times P_{\max}+(1-k)\times P_{\max}\times u$$ (20)

Proposed optimized model: Based on the objectives presented above, we present the optimization model according to the utilization and energy consumption. We assume that the datacenter contains *m* VMs *VM* = {*Vm*_1_,*Vm*_2_,…,*Vm*_*n*_} and *n* servers *PM* = {*Pm*_1_,*Pm*_2_,…,*Pm*_*n*_}. The issue to be solved is the VM placement problem. In general, we assume that more VMs are placed in fewer servers. We can then formulate the proposed optimization model. For example, the first purpose of the optimization is to minimize the SLA violations, as listed in the Eq 21, in which *S*_*i*_ represents whether at the current host is selected or not. The second purpose is to reduce the resource wastage, which is formalized as Eq 22, in which *U*^*p*^ and *U*^*m*^ represent the CPU utilization and the memory utilization, respectively. The third purpose is to minimize the power consumption, which is given in Eq 23, in which *k* is the idle power consumption coefficient and is equal to 0.7, and *P*_max_ is the power consumption at full utilization. The parameter *V*_*i*_ shows whether the virtual machine is selected or not. *U*^*v*^ represents the CPU utilization of the virtual machine. Constraints 24 and 25 are responsible for the capacity constraints of the server, such as CPU and memory capacity. Constraint 26 shows whether the server or VM is selected or not. When the server is selected, it is equal to 1; otherwise, it is equal to zero. It is difficult to solve the multiobjective optimization problem. Here, we use the Pareto efficiency to minimize the SLA violations and energy consumption. Additionally, Lemma 2 states the Pareto efficiency, which is described in detail as follows.

$$Minimize\sum_{i=1}^{n}S_{i}•SLA$$ (21)

$$Minimize\sum_{i=1}^{n}W=\sum_{i=1}^{n}\left[S_{i}\times \frac{|U^{p}-U^{m}|}{\sqrt{{\left(U^{p}\right)}^{2}+{\left(U^{m}\right)}^{2}}}\right]$$ (22)

$$Minimize\sum_{i=1}^{n}P=\sum_{i=1}^{n}\left[S_{i}\times (k\times P_{\max}+(1-k)\times P_{\max}\times \frac{\sum_{i=1}^{m}\left(V_{i}•U^{v}\right)}{\sum_{i=1}^{m}V_{i}})\right]$$ (23)

$$\begin{array}{l} Subject\;to:\\ \sum_{i=1}^{n}S_{i}•U^{p}\leq R^{cpu} \end{array}$$ (24)

$$\sum_{i=1}^{n}S_{i}•U^{m}\leq R^{mem}$$ (25)

$$S_{i},V_{i}\in \{0,1\}$$ (26)

**Lemma 2**. During the scheduling process, we consider solving the optimization problem with *n* objectives and *m* solutions. The multiobjectives are the SLA violations *k*_1_, resource wastage *k*_2_ and energy consumption *k*_3_. The solution is kept as the dominated solution when it is not worse than the others in every objective. Eq 27 is listed as below.

$$Min\;f(K)=\{f_{1}(k_{1},k_{2},\ldots ,k_{n}),\ldots ,\;f_{m}(k_{1},k_{2},\ldots ,k_{n})\}\;Subject\;to:\;k\in \{k_{1},k_{2},k_{3}\}$$ (27)

This process is implemented in detail by using the following fast nondominated algorithm, which is used as the global search to obtain the optimal solution.

- Step 1: First, all of the solutions are evaluated.
- Step 2: Then, solutions *m*_1_ and *m*_2_ are compared according to the three metrics, including the SLA violations *k*_1_, resource wastage *k*_2_ and energy consumption *k*_3_.
- Step 3: If solution *m*_1_ is better than *m*_2_ for the proposed metrics, it is marked as the dominated solution.
- Step 4: Otherwise, it is marked as the nondominated solution.
- Step 5: All of the solutions are searched until reaching the end.

### Description of the improved ACO algorithm

This algorithm is a type of multiobjective scheduling approach, which considers minimizing the SLA violations, resource wastage and energy consumption. In this approach, we use the ACO algorithm to obtain reasonable results based on behavior probabilities. In addition, the proposed ACO algorithm attempts to select the proper positions to place the selected VMs. The feasible solution is achieved by selecting the suitable PM to place the VMs by using the multiobjective method. First, the ants choose a path randomly. They then target the position by using the fitness function *Fit*_*best*_ based on maximizing the utilization and reducing the energy consumption. Second, the pheromone is calculated and updated. Then, by using the Pareto efficiency, we compare all of the solutions to determine the dominated solution by minimizing the SLA violations and energy consumption solution. The detailed resource scheduling pseudocode is described in Algorithm 2. To implement the ACO algorithm, it is necessary to consider three main factors: the fitness function model, the pheromone and behavior probabilities. These factors are described in detail below.

Fitness function: When the ant travels, it forms the feasible solutions. To solve the problem efficiently, the fitness function is set by maximizing the utilization and minimizing the resource wastage and the energy consumption. Eq 31 is shown below. The parameters *α*, *β* and *γ* are the weight factors for the SLA violation, resource wastage and energy consumption, respectively. To maximize the utilization, the SLA violation function *fit*_*sla*_ is set based on the CPU utilization. When the utilization is higher than 0.8, fewer SLA violations occur. In Eq 28, when the utilization decreases, *fit*_*sla*_ increases. To minimize the resource wastage, it considers both the CPU and the memory. In Eq 29, when the resources are utilized fully, *fit*_*w*_ avoids wastage. The strategy to reduce the energy consumption is formalized in Eq 30. When the utilization increases, *fit*_*p*_ will increase. Based on Eqs 28–30, we define the fitness function *Fit* in Eq 31, in which it is necessary to determine the weight factors. By using the AHP method, we take weight factors *α*,*β*,*γ* as 0.39, 0.35 and 0.26, respectively. The higher the fitness value is, the more efficient a solution we obtain.

$$Maximize\;fit_{sla}=\frac{1}{1+e^{\left(U_{cpu}-0.8\right)}}$$ (28)

$$Maximize\;fit_{w}=\frac{|U_{cpu}-U_{mem}|}{\sqrt{{\left(U_{cpu}\right)}^{2}+{\left(U_{mem}\right)}^{2}}}$$ (29)

$$Maximize\;fit_{p}=\frac{U_{cpu}•(P_{idle}+(P_{\max}-P_{idle})•U_{cpu})/P_{\max}}{P_{\max}}$$ (30)

$$Fit=\alpha \times fit_{sla}+\beta \times fit_{w}+\gamma \times fit_{p}$$ (31)

The pheromone: Generally, the proposed ACO algorithm considers two factors, the phermone matrix and the probability matrix. These two factors play important roles during the scheduling process. In this section, the pheromone is updated by Eq 32. In the initial phase, the pheromone is equal to the constant *C*. During the scheduling process, the pheromone matrix is updated by Eq 32, in which *ρ* is the pheromone evaporation factor, and $\Delta \tau_{iu}^{best}$ is the incremental gain. As the incremental gain increases, the solution becomes more feasible.

$$\tau_{iu}=(1-\rho )\times \tau_{iu}+△\tau_{iu}^{best}$$ (32)

In Eq 33, the increasing gain $\Delta \tau_{iu}^{best}$ depends on the set multiobjective fitness function *Fit*_*best*_ that considers the SLA, resource wastage and energy consumption. When the increasing gain is bigger, a better path is achieved by the pheromone.

$$△\tau_{iu}^{best}=\{\begin{array}{cc} Fit_{best} & if\;plan(VM_{i},PM_{u})\in path\\ 0 & otherwise \end{array}$$ (33)

Behavior probabilities: Another important factor is the probability to choose the suitable approach, which is calculated from the pheromone information. Eq 34 defines the behavior probability. In Eq 34, when more pheromone is left by the ants, the path becomes more feasible. In Eq 34, *η*_*iu*_ is the heuristic information, which is applied in the behavior probabilities, and *α* and *β* are the weight factors of the pheromone and heuristic information, respectively.

$$P_{iu}^{k}(t)=\{\begin{array}{cc} \frac{\tau_{iu}^{\alpha }\times \eta_{iu}^{\beta }}{\sum_{s\in allow_{k}}\tau_{iu}^{\alpha }\times \eta_{iu}^{\beta }} & i\in allow_{k}\\ 0 & otherwise \end{array}$$ (34)

The heuristic information *η*_*iu*_ is determined by Eq 35. The heuristic information is identified by the sum of the variance distances. When the heuristic information is higher, the probability will be greater.

$$\eta_{iu}^{}(t)=\{\begin{array}{cc} \sum_{s\in allow_{k}}Variance(i,u) & i\in allow_{k}\\ 0 & otherwise \end{array}$$ (35)

After the parameters are determined by Eqs 32–35, the proposed ACO algorithm is implemented as shown Algorithm 2.

**Algorithm 2: The proposed ACO algorithm**

**Input**: the selected *VM* = {*Vm*_1_,*Vm*_2_,…,*Vm*_*k*_}

**Output**: map the selected VMs to the reasonable host

//First, each ant is initialized.

1. Initialize all of the ants

2. Select the path randomly

//Then, the iterations start.

3. While (*iter < iter*_*max*_)

4.    For each ant

//Start the evaluation

5.        Evaluate the fitness function by using Eqs 32–33

6.        if the fitness value is higher,

7.            the fitness value is taken as the current value

//The pheromone is updated.

8.                Update the pheromone

9.        End if

//The probability is updated.

10.                Select the path of behavior probabilities by using Eqs 34–35

11.            End For

12.        Until all of the VMs are placed in the hosts

13.        End iterations

14.        In the global search, compare all of the solutions of the fast nondominated algorithm according to the Pareto efficiency by minimizing the energy consumption, the SLA violations and the resource wastage

15.        End

## Experiments

Two types of experiments are designed in this paper. One is a simulated experiment, and the other is a set of real application request experiments. These experiments were implemented on the CloudStack platform to verify the validity of the proposed algorithm. The results demonstrate that the proposed algorithm improves not only the CPU utilization and the memory utilization and also reduces the SLA violation and energy consumption.

### Experiment settings

In the real-world testing experiments, we implemented the proposed algorithm with 7 PMs. One PM is installed on the CloudStack platform, and the other six use XenServers running in the management nodes (2.20 GHz Intel(R) Xeon(R) 8 CPU, 8 G of memory, running CenOs 6.9). We create 18 VMs (1 VCPU, 1 G memory, running CenOs 6.9) in the cluster. In addition, we divide the experimental settings into three parts. First, to evaluate the approach, we implement it under different workloads. Second, to analyze the performance, we define the performance metrics, including the SLA violation ratio, the energy consumption ratio and the resource wastage ratio. Then, to evaluate the proposed algorithm efficiently, we implement it by using other heuristic algorithms for comparison.

**Fig 3:**
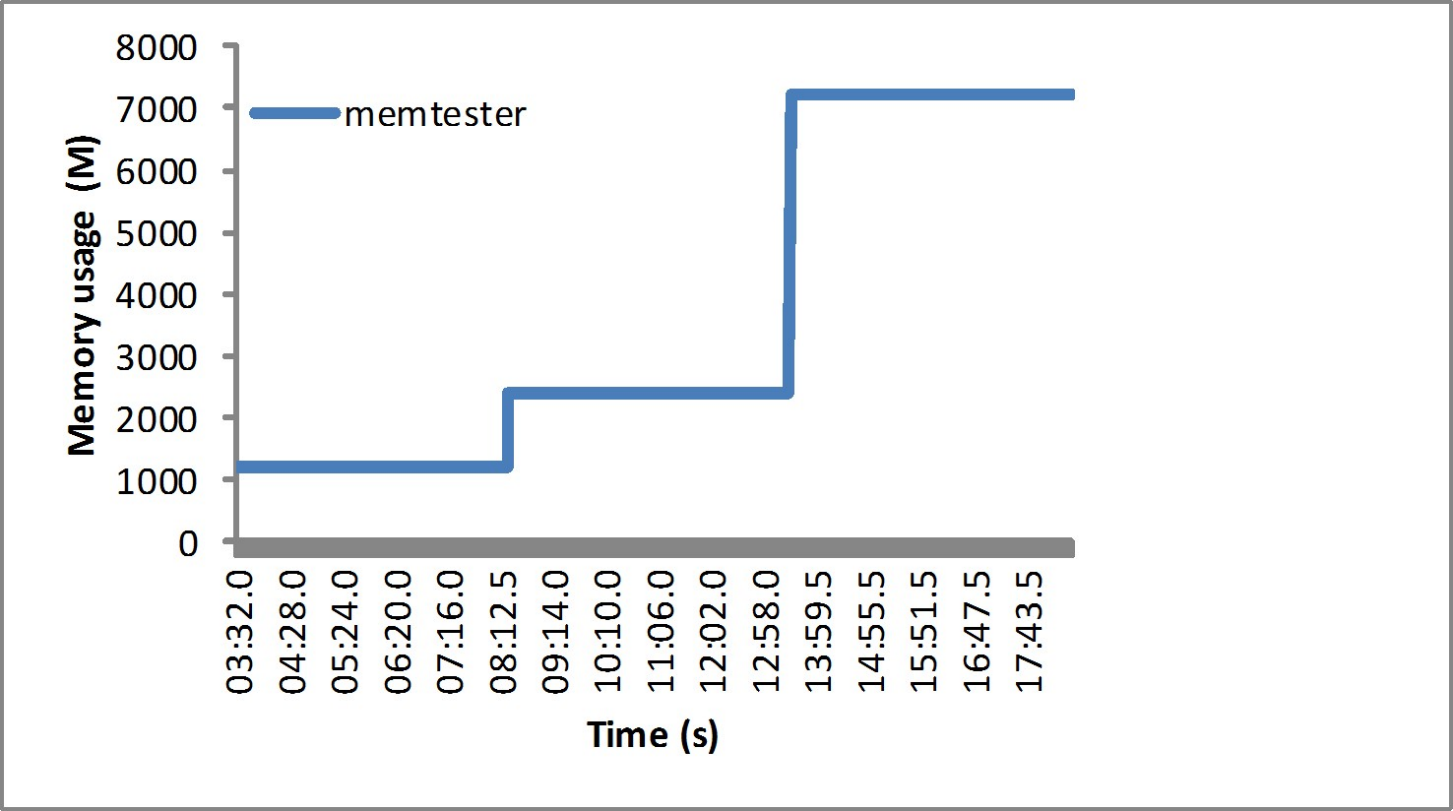
The designed load from the memtester.

**Fig 4:**
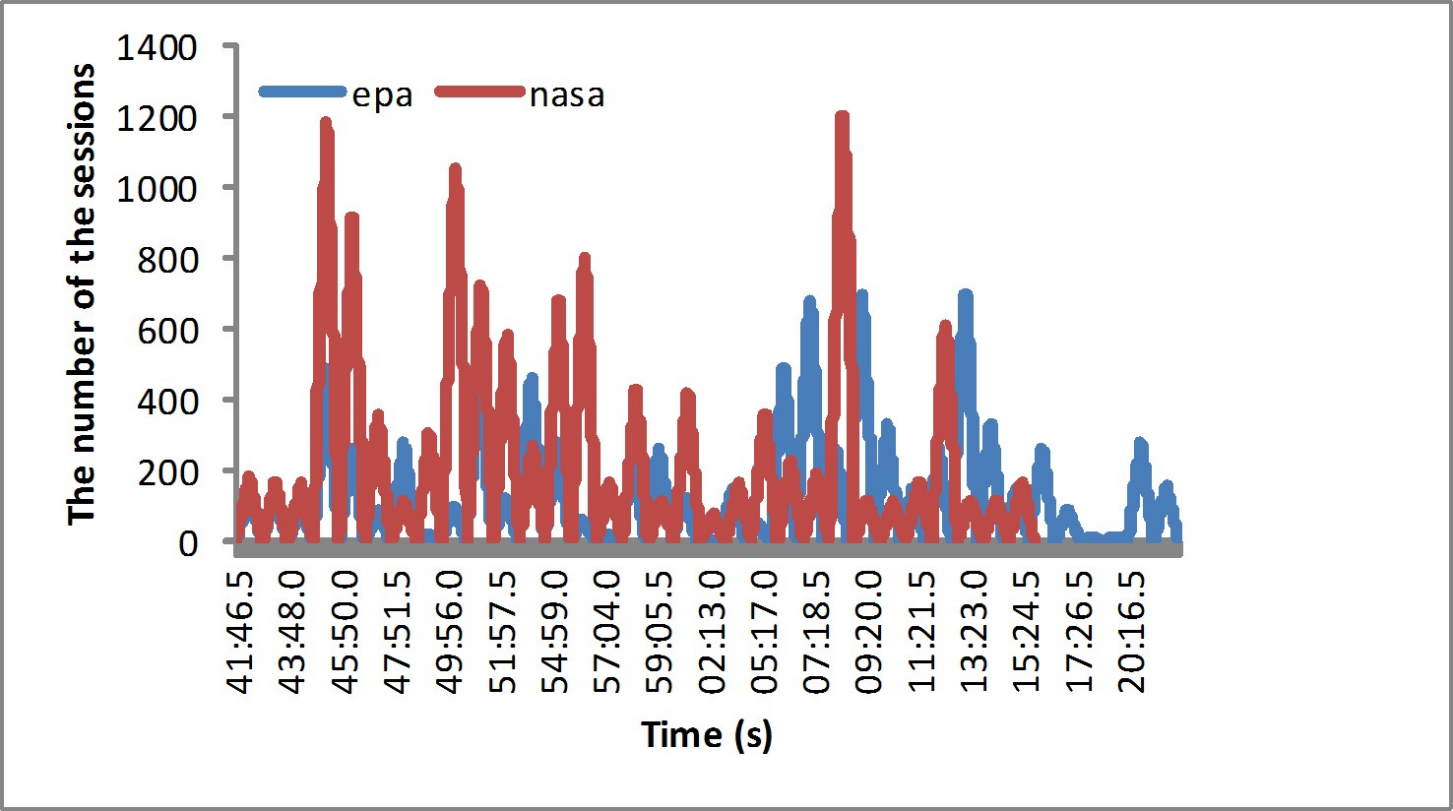
The simulated realistic workloads.

Experiment environment: To verify the proposed algorithm, we set two kinds of scenes in the experiments. In the first experiment, we used the workload generator memtester [50] to generate the CPU and memory workloads gradually, which is shown in Fig 3. In the second set of experiments, the application requests are generated by the TPC-W benchmark [51]. The second set of experiments implements the workload traces from real web requests, such as those from the EPA [52] and NASA [53]. We use Jmeter to generate the simulated application requests, as shown in Fig 4. The Jmeter plugin monitors more parameters, such as the CPU utilization and the memory utilization.

Metrics: The experiments use four performance indicators as metrics, such as SLA violation ratio *sla*_*ratio*_, resource utilization *U*_*cpu*_, energy consumption ratio *p*_*ratio*_ and resource wastage ratio *w*_*ratio*_. We then define the SLA violation ratio in Eq 36, which is the feedback of the requests to verify the SLA violations. The parameter *U*_*cpu*_ represents the CPU utilization of the server. When the CPU utilization is greater than 0.8, it may be easier to fall into an SLA violation. The percentage of the energy consumption is defined in Eq 37. The percentage of resource wastage is defined in Eq 38.

$$sla=\frac{1}{1+e^{\left(U_{cpu}-0.8\right)}},\;sla_{ratio}=\frac{\sum_{i=1}^{k}sla_{i}}{total_{sla}}$$ (36)

$$p_{ratio}=\frac{\sum_{i=1}^{k}P_{i}}{total_{P}}$$ (37)

$$w_{ratio}=\frac{\sum_{i=1}^{k}W_{i}}{total_{W}}$$ (38)

In these equations, *k* represents the number of the servers, *P*_*i*_ and *W*_*i*_ are the current power and resource wastage percentages, respectively. *total*_*w*_ and *total*_*P*_ are the total resource wastage and the total energy consumption, respectively.

Comparison of the algorithms in comparison: To validate the proposed algorithm, we compare it with other algorithms from the perspectives of minimizing the energy consumption and the SLA violations. The other algorithms are listed in detail below.

- Single objective algorithm: In [54], the single objective approach consolidates the resources from the perspective of minimizing energy consumption. It is implemented by using the ACO algorithm. The other goal is to maximize the utilization by using a bin-packing algorithm during the resource allocation, such as the FF (first fit) algorithm.
- Double-objective algorithm: In [55], the authors consolidate the resources based on two objectives, such as energy consumption and resource wastage. The proposed algorithm is implemented by using the ACO algorithm.
- Multiobjective algorithm: In [56], the authors proposed the ACO algorithm to consolidate the resources based on multiple objectives, such as power consumption and SLA violations. However, it regards these parameters with the equal weights, but the parameters are slightly different. We propose an improved multiobjective algorithm based on the proposed ACO algorithm, which considers the SLA violations, resource wastage and energy consumption with different weight factors by using the AHP method.

These experiments were performed across the cloud platform. A comparison of other algorithms shows the accuracy of the proposed algorithm. We also consolidate resources efficiently by using the multiobjective method. The experiments use 100 ants and 100 iterations, and the parameters *α* and *β* are set to 2 and 3, respectively, in the experiments.

### Experimental results

To verify the performance of the proposed algorithm, we compare it with other algorithms, including the single objective algorithm (ACO-U), the double-objective algorithm (ACO-UP) [55] and the multiobjective algorithm (MACO) [56]. MPSO is similar to the MACO algorithm; they use the same fitness function, but the MSPO is implemented by using the PSO algorithm. ACO-U is similar to the FF (first fit) algorithm, which puts the VMs into the PM of the minimal utilization. The experiments are designed with two types of workload; a synthetic load and a realistic load. The proposed algorithm attempts to simultaneously minimize the resource wastage and reducing the SLA violations and energy consumption.

Sythetic loads: The designed workloads are composed of three groups. Group 1 represents the lower variability workload, with the memtesters running in two VMs at nearly full utilization. Group 2 proposes slightly higher variability workload, with the memtesters running in four VMs at nearly full utilization respectively. Group 3 proposes a greater variability workload, with the memtesters running in eight VMs at nearly full utilization. The proposed algorithm is evaluated by four metrics: SLA violation ratio, resource utilization, energy consumption and resource wastage ratio.

**Fig 5:**
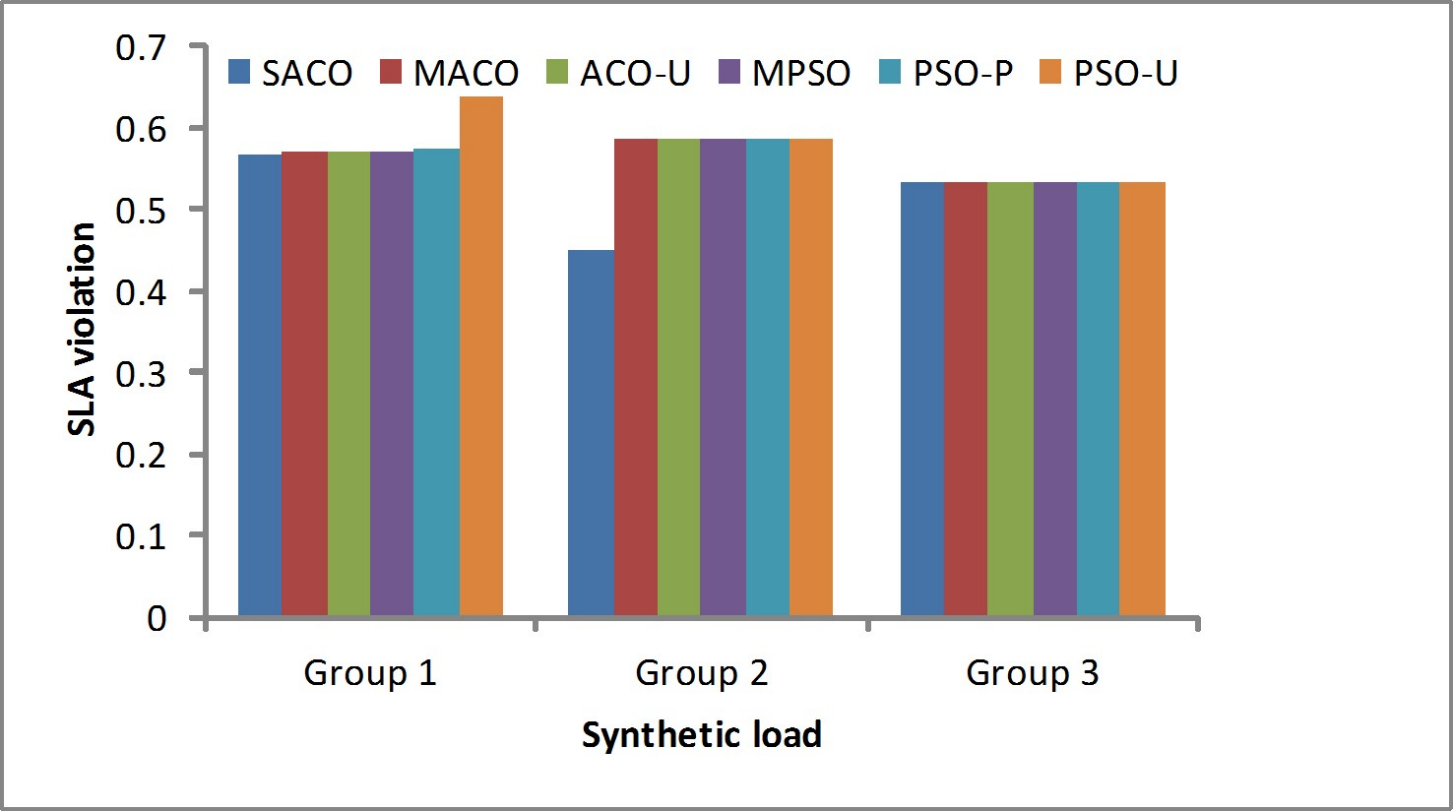
SLA violation rates under the synthetic loads.

SLA violation ratio: The SLA violation rate is one of the performance metrics. Fig 5 shows the SLA violation ratio for the six methods. The proposed algorithm (SACO) is clearly the best from the perspective of the SLA violations, such as multiobjective ACO (MACO) and multiobjective PSO (MPSO). In addition, the purpose of the ACO-U algorithm is to maximize the utilization to reduce the rental cost. This is the same purpose as the PSO-U algorithm. The intent of the PSO-P algorithm is to reduce the energy consumption. However, when the workload increases, the SACO algorithm has advantages and disadvantages; it can achieve the fewer SLA violations than the other algorithms for Group 1 and Group 2, but when the workload is higher, it is nearly the same as the other algorithms.

**Fig 6:**
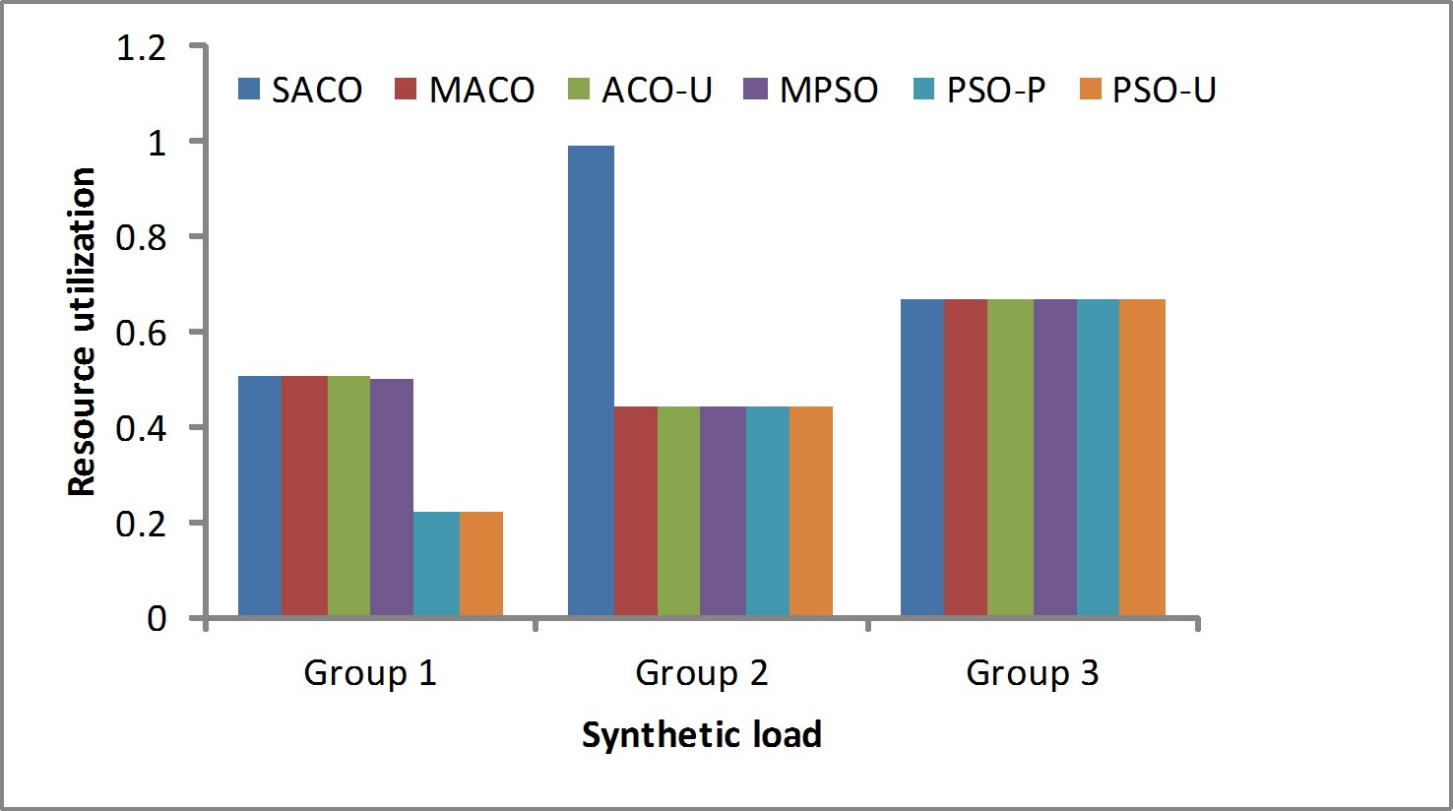
CPU resource utilization under the synthetic loads.

Resource utilization: The purpose of the resource utilization is to maximize the utilization to reduce the rental cost. Fig 6 shows that the SACO algorithm is better than the other algorithms. When the workload is light (Group 1), it achieves the better consolidation results than the other algorithms. When the workload is moderate (Group 2), the proposed solution is superior to the other algorithms; improves the resource utilization and reduces the rental cost. When the workload is heavy (Group 3), the proposed algorithm is approximately the same as the other algorithms. In other words, the results for these three groups are reasonable and demonstrate the effectiveness of the SACO algorithm.

**Fig 7:**
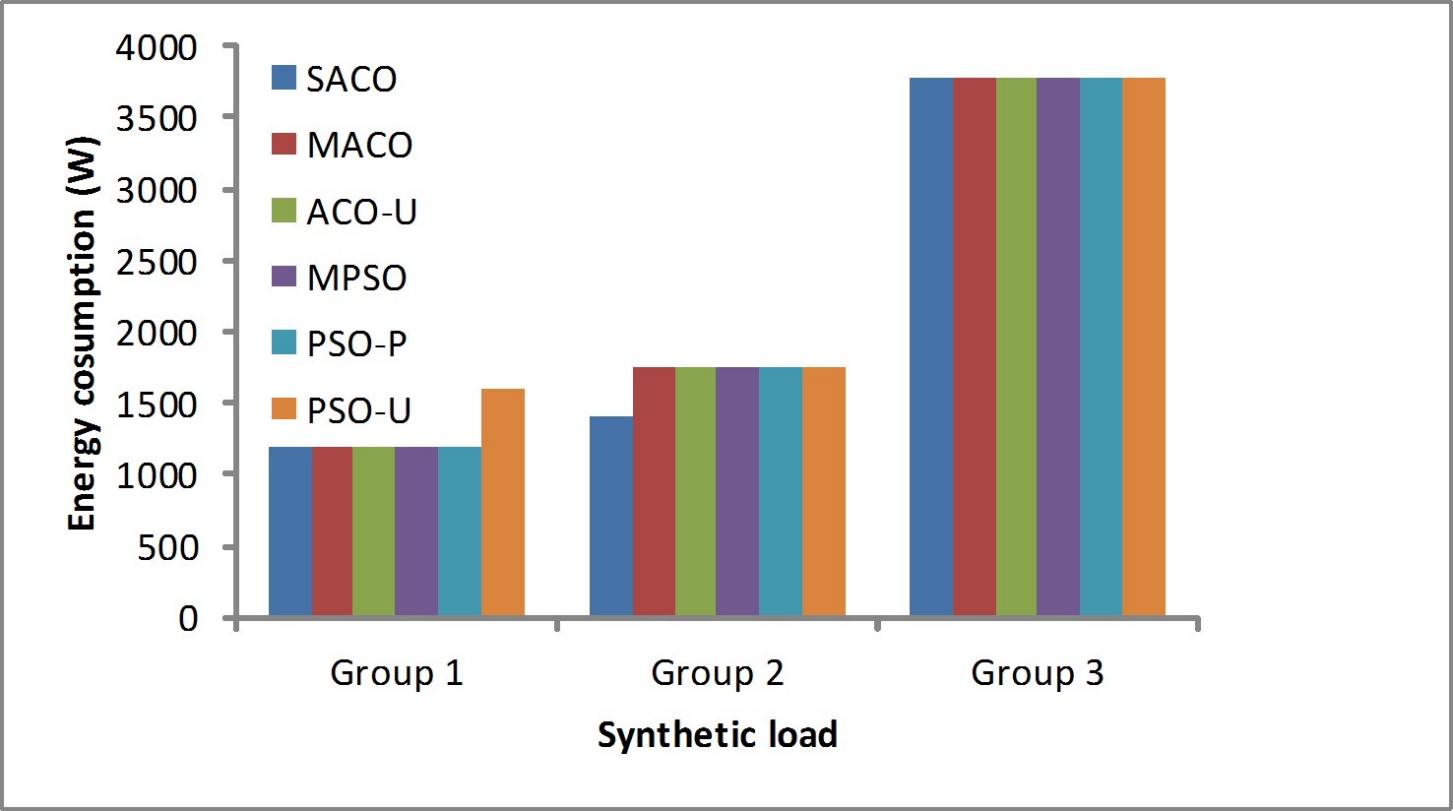
Energy consumption under the synthetic loads.

Energy consumption: A solution to reduce energy consumption is necessary. As shown in Fig 7, the SACO algorithm obtains the better results for minimizing energy consumption. With the lighter load (Group 1), the SACO algorithm achieves slightly better results than the other algorithms. With the higher workload in Group 2, the SACO algorithm is better than the other algorithms because it considers multiple objectives by using the Pareto policy. When the generated load in Group 3 is implemented, the performance is nearly to the same as the other algorithms. Generally, the SACO algorithm is reasonable for the synthetic loads, and it gives the better results during the scheduling process.

**Fig 8:**
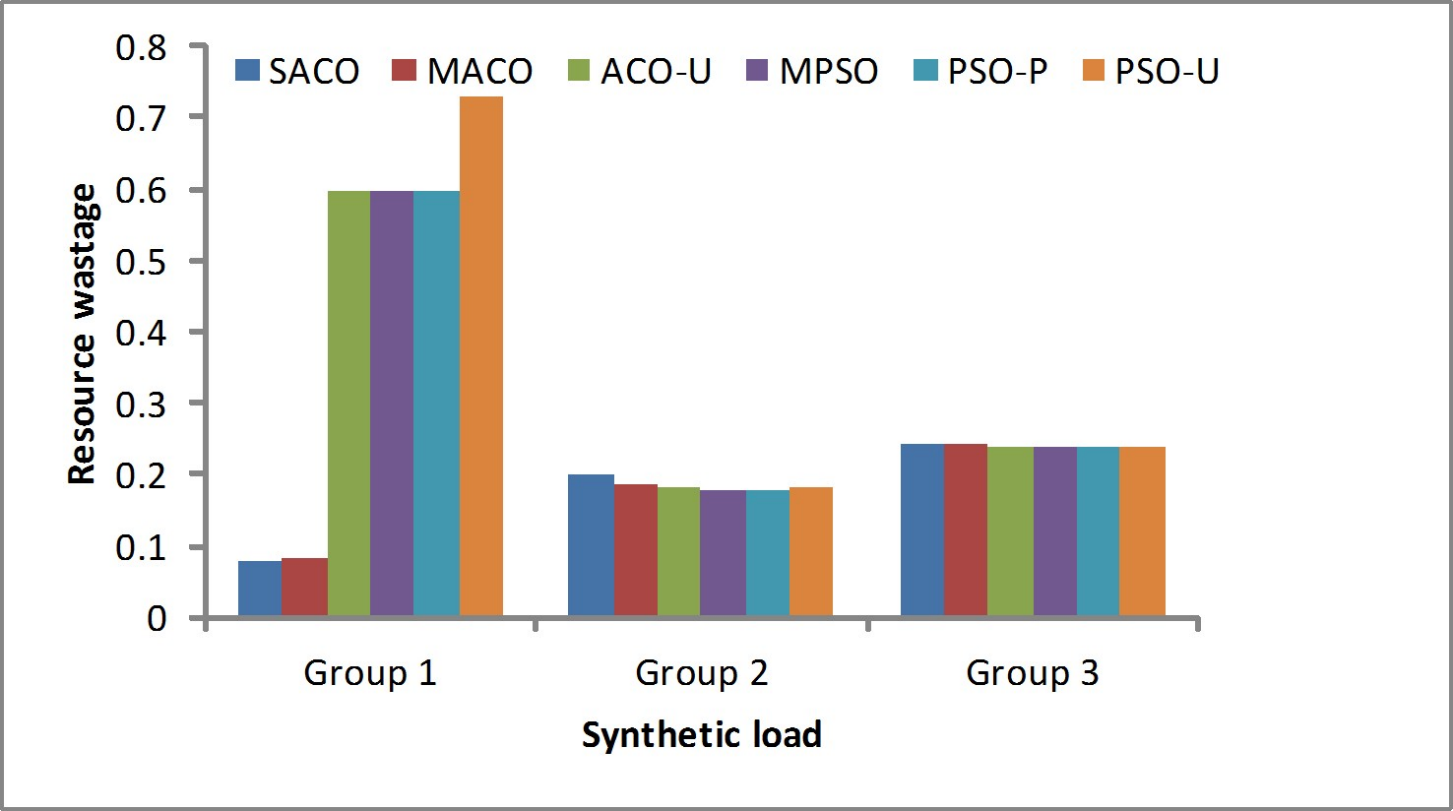
Resource wastage rates in the synthetic loads.

Resource wastage ratio: The resource wastage is the metric used to measure the degree of resource wastage. This experiment verifies the degree of resource wastage by comparisons with other methods. The SACO algorithm proposed a resource wastage model that includes the CPU utilization and the memory utilization, which is designed according to Eq 19. The other algorithms focus more on the CPU utilization. Fig 8 shows that the SACO algorithm gives better results. It achieves the lower resource wastage in Group 1, whereas in Groups 2 and 3, it is approximately to the same as other algorithms. This is because these six methods all consider the CPU utilization similarly. However, the SACO algorithm obtains slightly better results than the other algorithms.

Realistic loads: The depicted loads are implemented by simulated real-world workloads, such as those from the EPA and NASA. In these two simulated real-world workload experiments, the EPA workload is taken as the lower variability workload. The NASA workload has a slightly higher variability. The results are analyzed by the metrics as follows. With the real workloads, the proposed approach is compared with other ACO algorithms based on one or multiple objectives. For example, the SACO algorithm is implemented by using the fixed fitness function of different weights. The MACO algorithm [56] takes the set fitness function with equal weights, and the weight factors are the same. The goal of the ACO-P algorithm [54] is to reduce the power consumption. The purpose of the ACO-UP algorithm [55] is to maximize the utilization and reduce the energy consumption. The experimental results are described in detail below.

**Fig 9:**
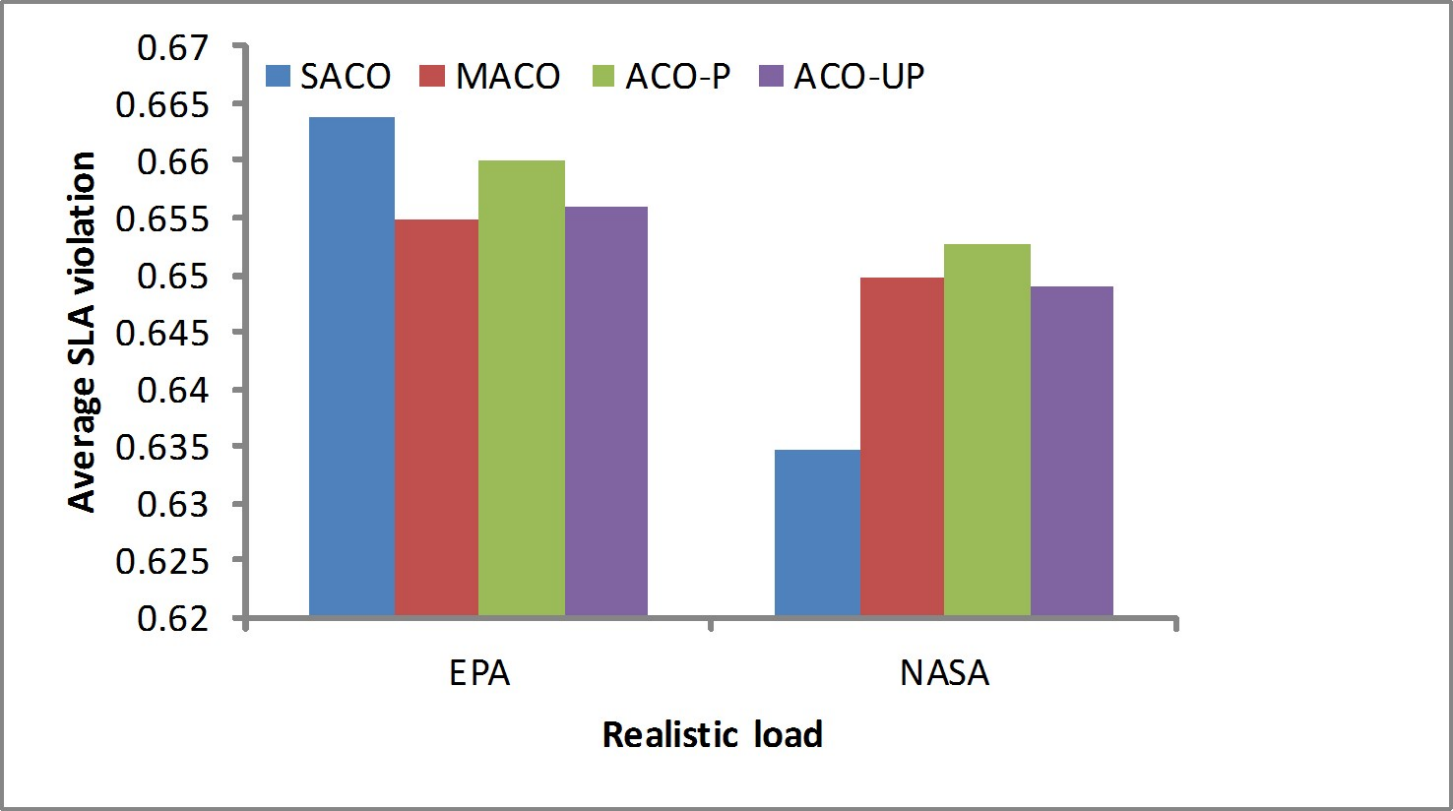
SLA violation rates under the realistic loads.

SLA violation ratio: The SLA violation is one of the performance metrics. In the EPA experiment, as shown in Fig 9, the SACO algorithm obtains slightly worse results for the SLA violation ratio than the other algorithms because it considers more elements when running under the lighter load. As shown in Fig 9, with the NASA workload, which is a heavier load, the results are better than those of the other algorithms. Therefore, the proposed algorithm is more suitable for minimizing the SLA violations under the heavy loads, such as the NASA workload.

**Fig 10:**
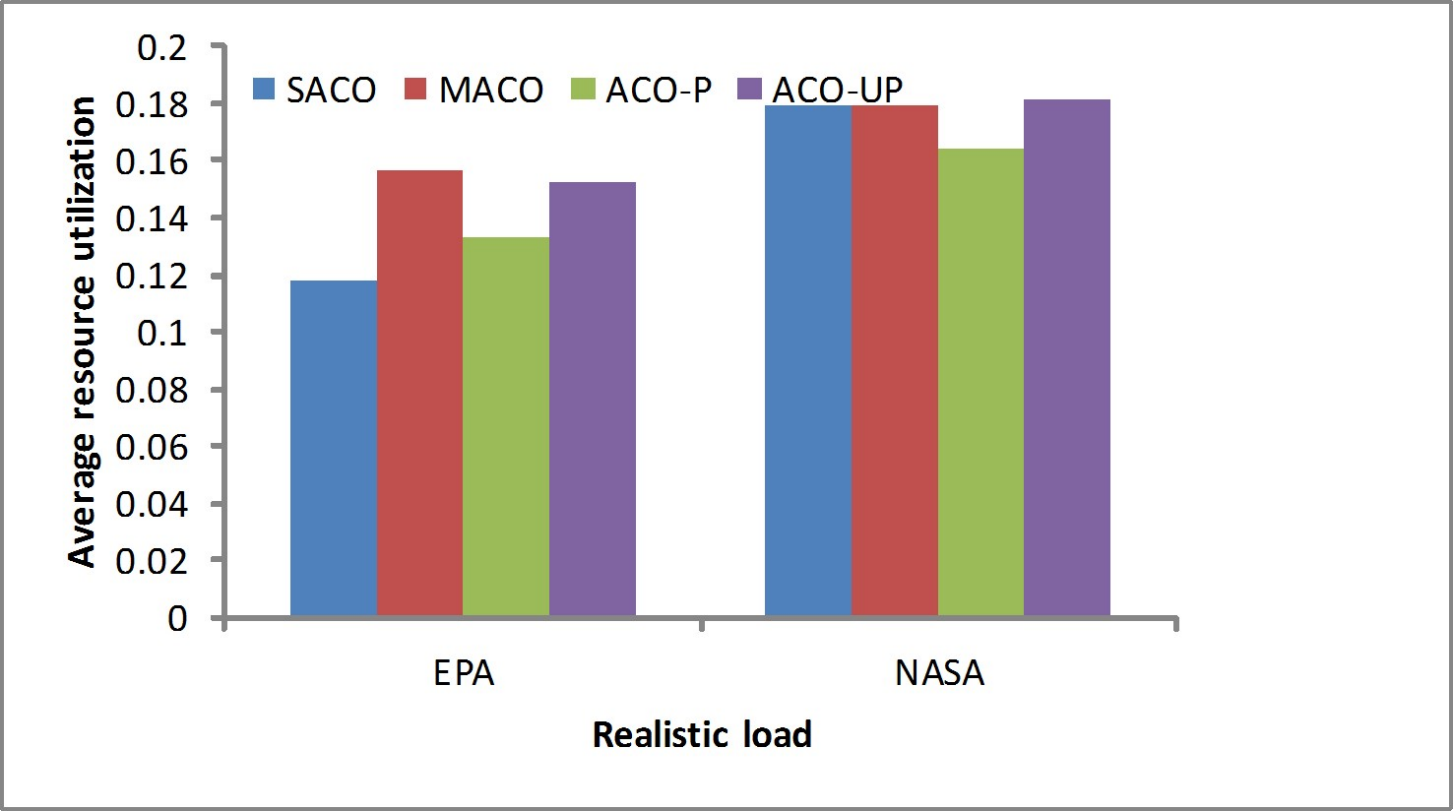
CPU resource utilization under the realistic loads.

Resource utilization: Usually, the consolidation algorithm focuses on maximizing the resource utilization and reducing the rental cost for the providers. As shown in Fig 10, we achieve slightly worse results with the EPA wrokload and better results with the NASAworkload. The proposed solution (SACO) is more efficient than the others for maximizing the resource utilization under the heavy loads, such as the NASA workload.

**Fig 11:**
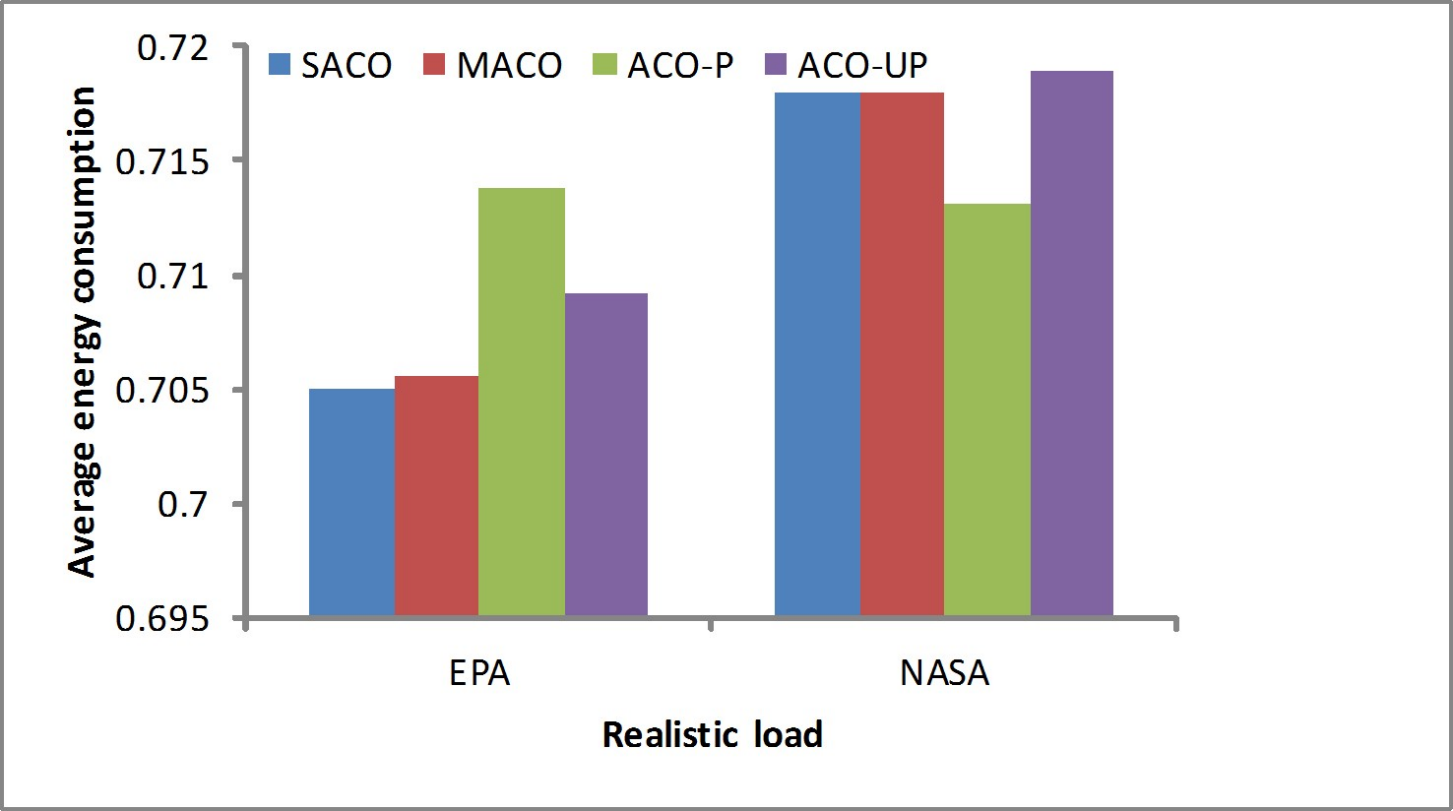
Energy consumption under the realistic loads.

Energy consumption: It is important to solve the energy consumption problem by choosing a suitable algorithm during the dynamic scheduling process. As shown in Fig 11, the SACO algorithm is clearly better than the others under the EPA workload. This is because it is easier to consolidate the resources when running under a lighter load. With the NASA workload, the SACO algorithm is slightly worse than the others. In addition, the ACO-P algorithm obtains the better results for minimizing the energy consumption under the NASA workload.

**Fig 12:**
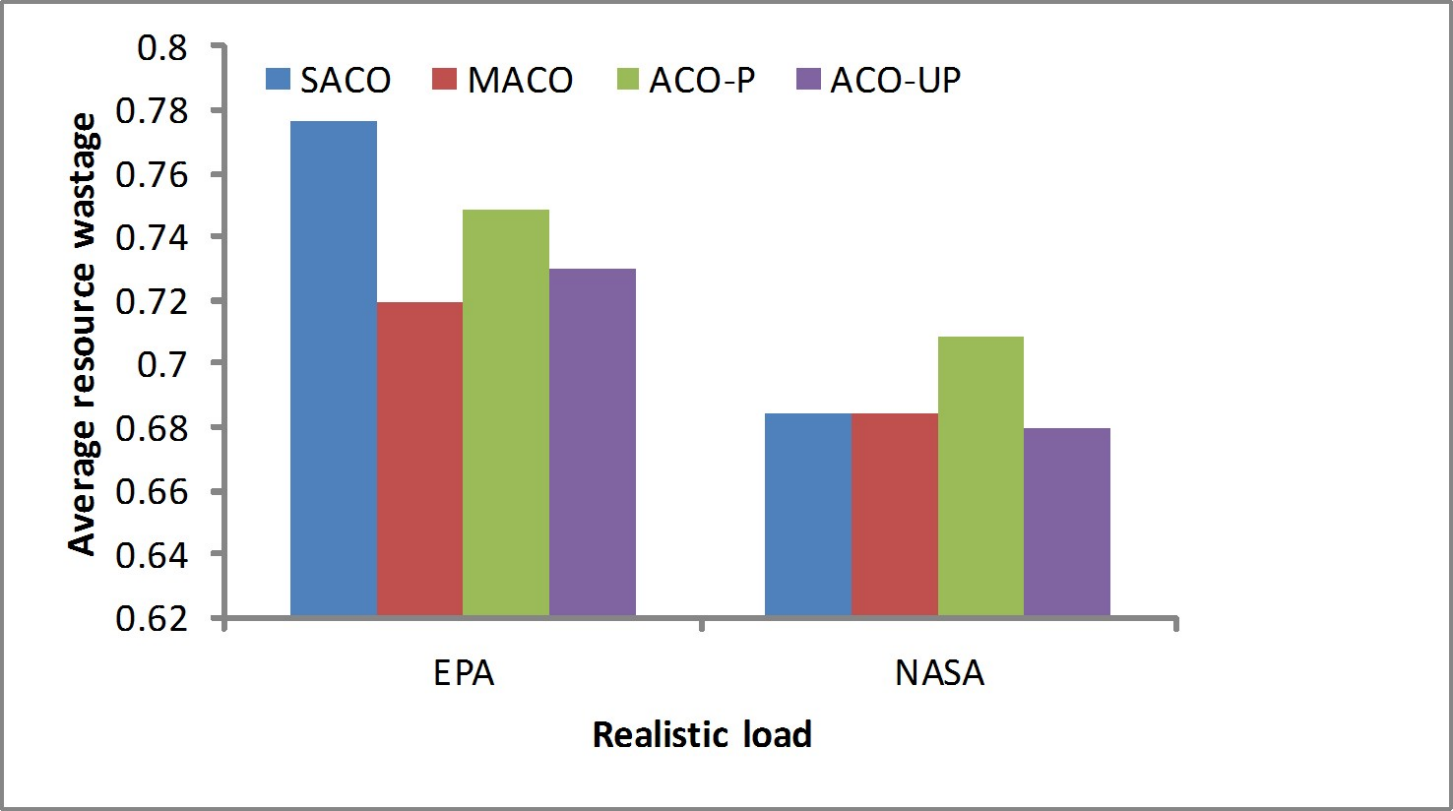
CPU resource wastage rates under the realistic loads.

Resource wastage ratio: The degree of resource wastage degree is an indicators of whether the resources are used efficiently. As shown in Fig 12, the SACO algorithm obtains slightly worse results than the others under the EPA workload, and it obtains the better results than the others under the NASA workload. The proposed solution considers more factors. Therefore, it is clear that the SACO algorithm is more efficient under denser loads, such as the NASA workload.

In summary, realistic workloads were analyzed using simulated real-world loads, such as the EPA and NASA workloads. The results show that the SACO algorithm slightly outperforms the others because it considers more factors, such as the SLA violations, resource wastage and energy consumption. In addition, it provides more efficient solutions for realistic loads. A summary of the results from the realistic loads is given in Table 3.

**Table 3:** The results of the realistic workload by methods.

Objectives	EPA			NASA		
	SLA	Resource utilization	Power	SLA	Resource utilization	Power
ACO-P	0.660	0.133	0.713	0.652	0.163	0.713
ACO-UP	0.655	0.152	0.709	0.649	0.181	0.718
MACO	0.654	0.156	0.705	0.649	0.179	0.717
SACO	0.663	0.117	0.704	0.634	0.179	0.717

## Conclusion

Traditional scheduling approaches focus on the energy model to reduce the overhead. However, additional factors have effects during the scheduling process. In this paper, we develop a novel consolidation algorithm that uses multiple objectives, such as minimizing the cost overheads [57–58] and the power consumption [59]. *First*, we determine the hotspots by using the score model in the data center. When the score threshold reaches a specific value, the hotspots are identified. The score model solves the issue of when to migrate. *Second*, we quickly migrate the VMs by using the PSO algorithm. To save the energy overheads, we take the VMs in the under provisioning into the migrated list. This solves the question of which VMs should be migrated. *Third*, we propose an improved ACO algorithm that simultaneously attempts to minimize the rental cost and the power consumption. Using the Pareto efficiency leads to better quality in solving the resource consolidation problem. This solves the issue of where to migrate. We can then shut down the idle nodes and minimize the number of nodes. Finally, we evaluate the algorithm under simulated and real workloads. The results show that the proposed consolidation technique improves the utilization and enhances the scalability.

To enhance the depth of this study, further research will focus on several aspects. First, additional factors have influences on the dynamic scheduling problem, such as the temperature and the frequency of the CPU. Second, the scheduling algorithm can be applied in complex environments, such as for scientific workflows in IaaS. Degraded performance is another future research direction. For example, over long periods of time, data corruption and exhaustion of resources can cause performance degradation. Finally, the energy overhead should be investigated in detail, potentially by using the adaptive DVFS technique or cooling systems to manage the temperatures.

## Supporting information

S1 Table Synthetic load by the memetester. The ladder workload is generated by the memtester.
(XLSX)

S2 Table Real-world load by the Jmeter. The simulated real workload is generated by the Jmeter, such as the EPA and the NASA.
(XLSX)

S1 Performance Evaluation The SLA violation, the utilization, energy consumption and the resource wastage are evaluated under the synthetic load and real workloads. (XLSX)
